# Utilization of Psychiatric Team Driven Ketamine Infusions

**DOI:** 10.1192/j.eurpsy.2022.1435

**Published:** 2022-09-01

**Authors:** M. Ithman, B. Sobule, A. Campbell

**Affiliations:** University of Missouri-Columbia , Psychiatry, Colubmia, United States of America

**Keywords:** Ketamine, Suicidal Ideations, intervention, Depression

## Abstract

**Introduction:**

In 2018 Missouri University Psychiatric Center, an inpatient psychiatric hospital established a ketamine infusion team to treat severely depressed and acutely suicidal clients. Over 80 infusions were delivered over three years, with positive outcomes and minimal side effects.

**Objectives:**

To evaluate outcomes of an inpatient psychiatric intravenous ketamine team to deliver treatment without anesthisia colloabration, which could open the horizon for future intravenous medications in a psychiatric inpatient setting.

**Methods:**

A team consisting of a psychiatrist supported by a psychiatric PA, psychiatric pharmacist, and a mental health nurse devloped a protocol including physical and mental health screening, inclusion/exclusion criteria, dosing, and client monitoring. For data collection, the team monitored vital signs and mental status changes for tolerability and depression screening tools for efficacy.

**Results:**

**Table 1:** Ketamine Infusion Data

Total Clients	32
Male	15
Female	17
Dosing	0.5mg/kg adjusted bodyweight infused over 40 minutes
Average Baseline Depression Screening (PHQ or QIDS)*	20.8
Average Baseline Follow up Screening (PHQ or QIDS)*	7.5
Average Change in Screening Score (PHQ or QIDS)*	-14.1
% Change From Baseline in Screening Score (PHQ or QIDS)*	65.5%
Adverse Events Documented	Dissociation 5 (15.6%); Nausea/Vomiting 3 (.09%), Extreme Euphoria 2 (.06%); sedation 1 (.03%); BP Increase 1 (.03%)

**Conclusions:**

Overall, ketamine infusions were tolerated well with limited adverse drugs reactions reported or observed and were easily addressed by the team without any serious adverse events. Given the rapid improvement of symptoms and overall tolerability, intravenous ketamine infusions conducted solely by a psychiatric-based team advances our field for further treament modalities.

**Disclosure:**

No significant relationships.

